# Functionalized Polymers for Enhance Oral Bioavailability of Sensitive Molecules

**DOI:** 10.3390/polym8060214

**Published:** 2016-06-02

**Authors:** Yolanda Alvarado Pérez, Claudia Muro Urista, Javier Illescas Martínez, María Del Carmen Díaz Nava, Francisco A. Riera Rodríguez

**Affiliations:** 1 Departamento de Ingeniería Química e Investigación, Instituto Tecnológico de Toluca, Apartado Postal 890, 52149 Metepec, MEX, Mexico; yoal68@hotmail.com (Y.A.P.); jillescasm@ittoluca.edu.mx (J.I.M.); cdiaz@ittoluca.edu.mx (M.D.C.D.N.); 2 Departamento de Ingeniería Química y Tecnología de Medio Ambiente, Universidad de Oviedo, Oviedo, 33006 Asturias, Spain; far@uniovi.es

**Keywords:** functionalized polymers, enhance strategies, biopharmaceuticals, oral bioavailability

## Abstract

Currently, many sensitive molecules have been studied for effective oral administration. These substances are biologically active compounds that mainly suffer early degradation in the gastrointestinal tract (GIT) and physicochemical instability, inactivation and poor solubility and permeability. The sensibility of the biomolecules has limited their oral administration in the body and today is an important research topic to achieve desired effects in medicine field. Under this perspective, various enhancement approaches have been studied as alternatives to increase their oral bioavailability. Some of these strategies include functionalized polymers to provide specific useful benefits as protection to the intestinal tract by preventing its degradation by stomach enzymes, to increase their absorption, permeability, stability, and to make a proper release in the GIT. Due to specific chemical groups, shapes and sizes, morphologies, mechanical properties, and degradation, recent advances in functionalized polymers have opened the door to great possibilities to improve the physicochemical characteristics of these biopharmaceuticals. Today, many biomolecules are found in basic studies, preclinical steps, and others are late stage clinical development. This review summarizes the contribution of functionalized polymers to enhance oral bioavailability of sensitive molecules and their application status in medicine for different diseases. Future trends of these polymers and their possible uses to achieve different formulation goals for oral delivery are also covered in this manuscript.

## 1. Introduction

A large number of proteins and peptides (vaccines, enzymes, hormones, cytokines, antibodies nucleic acids and globulins) have found possible applications as biopharmaceuticals because they can be used for the treatment of many diseases, such as diabetes (insulin), and malignant disorders, including cancer. As a result of their importance in the medical field, today many biopharmaceuticals make up about one-third of drugs currently in development [1]. However, they are sensitive molecules that are hardly administered in their oral form, because of their several unfavorable physicochemical properties, including large molecular size, susceptibility to enzymatic degradation, short plasma half-life, ion permeability, immunogenicity, and the tendency to undergo aggregation, adsorption, and denaturation [2]. The fast degradation of active substances in the gastrointestinal tract (GIT) and poor permeability across the intestinal epithelium should be improved to achieve appropriate concentrations in blood and to extend the residence time in the body; since to exert their health benefits, the active substances have to be delivered in the site of absorption. These factors, including stability and circulation time in the gastrointestinal tract, strongly affect the effective absorption of oral-delivered drugs, as well as the inability to reach their targets in an active form *in vivo* [3].

With the aim of reducing these constraints, some biopharmaceuticals are currently administrated by intravenous, intramuscular or subcutaneous route, although their effects are generally poor, since molecules cannot get to the active site in denatured way, or they are not allowed to cross the intestinal barrier. This problem severely restricts the therapeutic value of the drugs, particularly for diabetes, the case is more evident: less than 0.1% of the oral dose of insulin reaches the bloodstream intact [4]; thus, due to their excessive degradation, it must be administered through injections and in repeated doses to ensure their effect, which leads to poor patient compliance [5].

Other alternative routes to transport sensitive molecules include the buccal, intranasal, pulmonary, transdermal, ocular, vaginal and rectal routes. In particular, oral transmucosal buccal delivery and sublingual delivery have progressed far beyond the use of traditional dosage forms with novel approaches, which could be engineered to deliver complex biomolecules. Details on these routes are found in [6,7,8]; however, these alternatives have only had success in some cases, since both, the stability of the formulations and their preparation are the most important hurdles for their oral delivery.

Consequently, several studies have focused on the research of suitable administration of these molecules, but certainly, the increment in their oral bioavailability, adequate releasing in GIT and their half-lives, *in vivo*, are one of the great challenges in the field of medicine [9].

In general, successful oral delivery of drugs in GIT requires the accomplishment of three main aspects: (i) protection of the macromolecules from degradation; (ii) permeation through the intestinal barrier; and (iii) absorption into the systemic circulation [10,11].

Under these requirements, oral bioavailability of sensitive molecules is recognized according to the Biopharmaceutics Classification System (BCS) [12] as follows: (1) Class IV, drugs with low intestinal permeability and low solubility; they have a high degree of difficulty penetrating GIT and thus it is necessary to surpass these limitations. (2) Class III, drugs with poor intestinal permeability and high solubility, they require an important enhancement in their permeability. In this category, commercially relevant molecules include: insulin, glucagon-like-peptide 1 and analogues like salmon calcitonin, octreotide, parathyroid hormone and LHRH hormone analogues such as leuprolide. (3) Class II, drugs are concerning to macromolecules with permeability issues (high permeability and low solubility); include molecules unfractionated such as heparin, low-molecular weight heparins, antisense oligonucleotides and vancomycin. Finally, (4) Class I, the small molecules with high solubility-high permeability, belong to this category; among them, bisphosphonates, acyline, amphotericin, camptothecin and gentamycin can be found [13].

The BCS classification is considered a scientific criterion for establishing the status of oral bioavailability of drugs; it is based on their aqueous solubility and intestinal permeability properties. BCS leads to employ specific tests *in vitro* in order to predict the drugs dissolution and then to estimate the results of their bioavailability *in vivo*. This procedure has a significant impact on drug policy, making it possible to exempt bioavailability tests *in vivo* for class I, II and III drugs in oral solid dosage forms, which simplifies the registration of new medicines [14]. Currently, this criterion is accepted by different organisms, such as the U.S. Food and Drug Administration (FDA), the Brazilian Agência Nacional de Vigilância Sanitária (ANVISA) and the European Medicines Agency (EMA) FDA, ANVISA and EMA for biowaiver *in vivo* studies of sensitive molecules that have been studied under standard solubility and dissolution values by specific *in vitro* trials.

Due to the importance of this topic, many researchers and different specialized companies are found pending of the investigation and results on evolution status of the oral bioavailability of sensitive molecules. In this regard, various studies are focused in the application of different enhancement techniques to resolve the problems of the solubility and permeability of these substances. The techniques are based in physical or chemical modification of the molecule, such as: (1) Physical modification by incorporation of permeability enhancers or enzyme inhibitors, and use of carriers as protective and delivery systems, which can have different functions; for example, permeability enhancer, protection against degradation, stabilizer and/or molecule carrier. (2) Chemical modification of molecules, mainly to improve their lipophilicity, permeability and susceptibility to degradation [5,8,10].

Many enhancement techniques comprise the application of benign biodegradable polymers, explicitly the functionalized polymers (FP). These materials are becoming increasingly important in specific application for oral bioavailability of drugs. FP contain polymers with specific chemical groups, having different biological and pharmacological applications. These groups provide novel and tailor-made properties out of existing polymers, as well as relevant advantages in drug delivery by targeting molecules in GIT. They also improve the aqueous solubility properties of the poorly aqueous soluble drugs, control drug release over time and improve the stability of therapeutic agents against enzymatic degradation [15].

FP systems include different arrangements and structures, such as, FP-molecule conjugates, molecule loaded block FP micelles and self-assembled or FP-based vehicles as microparticles or nanoparticles.

Standard polymeric materials such as polyolefins, polyesters, polyamides, cellulose, chitosan and nylon have been the most employed as base matrix, to obtain adequate FP in this area. The incorporation of synthetic polymers, such as poly(caprolactone) (PCL), poly(ethylene glycol) (PEG), poly(vinyl alcohol) (PVA), poly(acrylamide) (PAAm), poly(glycolic acid) (PGA), poly(lactic acid) (PLA) and poly(γ-glutamic acid) (PGA), as well as their functionalization by benzaldehyde (BA), Tetrathiafulvalene (TTF), vinil acrylic acid (VAA), boric acid (BAc) and phenolic acid (PhAc) have attracted much attention, since they can improve the properties of these FP systems. Some of these include biocompatibility, non-toxicity, no-allergenic properties and biodegradability. Meanwhile, the incorporation of active groups (hydroxyl or primary amine) in these polymers have been used to attach the molecules, achieving acceptable results like permeability enhancers, lipophilicity and protection against the nature of enzymes or as a system for controlled drug release.

This review summarizes physical and chemical alternatives based on FP systems to enhance oral delivery and bioavailability of sensitive molecules and their application status. The main approach of application of FP is described for different systems as the alternative delivery in the GIT. Some of these forms also include combined strategies of FP systems for their optimization in drug delivery.

## 2. Physical Modifications of FP to Enhance Oral Bioavailability of Sensitive Molecules

Currently, effective alternatives by physical modification of sensitive molecules are associated with FP materials. FP include selective, non-toxic and biodegradable polymers with reactive groups or responsive characteristics to enhance functional properties and oral bioavailability of these molecules. Because of these properties, FP may enhance their stability, prolong their activity and protect them from the harsh environments of the stomach, before releasing the drug into the more favorable regions of the GI tract, specifically, the lower regions of the intestine. Besides, they can also maintain intimate contact with mucus to ensure immediate absorption in the luminal fluid [5].

FP based on standard polymers such as chitosan, starch, gelatin, pectin, cellulose, carboxymethyl cellulose (CMC), PVA and poly(lactic*-co-*glycolic acid) (PLGA), have been the most studied for this purpose. Specifically, there are various properties from polymers that are exploited to modifie sensitive molecules by FP. Particularly, some of them may act as an effective permeability enhancer, because of their mucoadhesive or metal chelating ability. FP built by polymer combinations of PVA, PCL and PEG present these properties and they have been used as safer strategies to increase intestinal absorption of biomolecules [13]. Mucoadhesive FP may also increment paracellular permeability of water-soluble, low lipophilic and poorly absorbable molecules by enlargement of tight junction (TJ) adhering to the intracellular portion and allowing drug permeation [16].

Hydrophilic polymers like poly(vinyl pyrrolidone) (PVP) or PEG provided stability and solubility properties to sensitive molecules, since they act as solubility enhancement. Suitable proportions of these polymers could give, as result, FP materials to prolong molecule shelf-life and contribute also to stabilization against drug crystallization from the solid phase. On the contrary, other polymers such as poly(vinylpyrrolidone*-co-*vinyl acetate) (PVPVA) and polyvinyl acetate phthalate (PVAP), may be used to modify hygroscopic materials, since carbonyl oxygens present in both polymers are used as intermolecular H-bond acceptors [11].

Other properties like the antibacterial activity of chitosan and polycarbophil have also been exploited to eliminate bacteria that are prone to degrade sensitive molecules [17].

From these advantages and properties that offer FP materials, their study in the application as delivery systems of sensitive molecules has been possible. Currently, there are various forms of FP systems to enhance their oral bioavailability: micelles, liposomes, complexes and structures with shapes and sizes, with tailored pores, different morphologies and mechanical properties constitute these FP systems. In addition, hydrogels, micro and nano-emulsions, nanoparticles, platelet, core–shell structures, microspheres, pellets, dendrimers and films are some other types [18].

**Figure 1:**
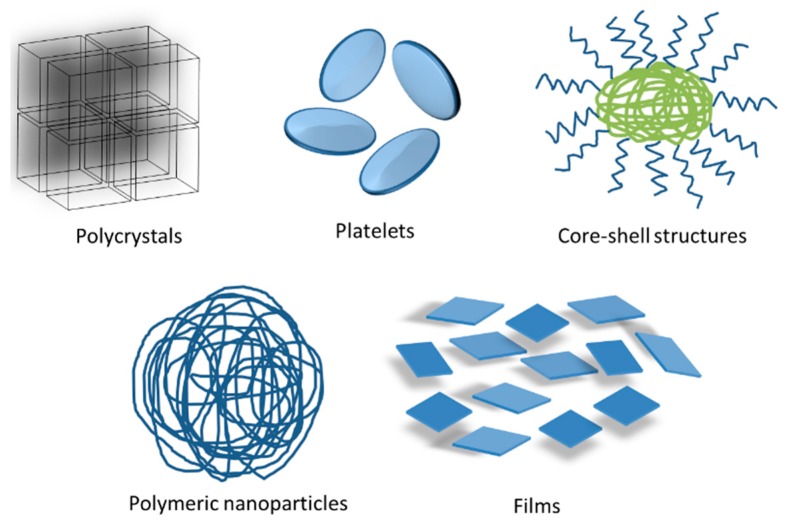
Different FP systems used to enhance the bioavailability of sensitive molecules.

Figure 1 shows the most studied FP systems for sensitive molecules delivery. Several FP are used as base materials to fulfill the purpose; such as, polysaccharides (chitosan, starch and cellulose derivatives); proteins (gelatin, soy, casein and whey), poly(acrylic acid) (PAAc), glycerol, PEG and various gums such as guar, xanthan, PVP and PVA [19,20].

**Figure 2:**
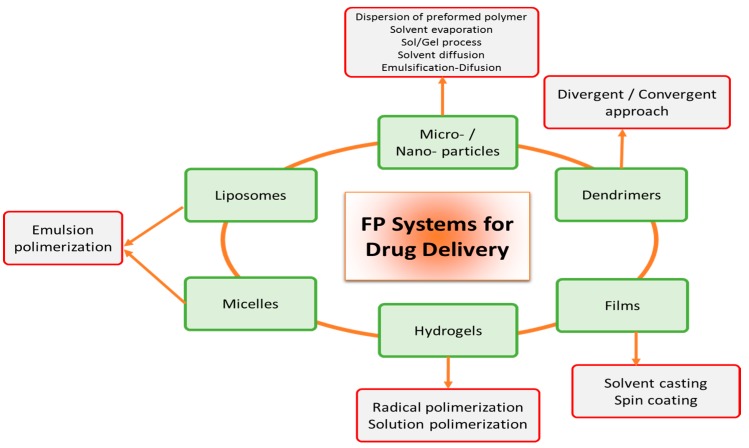
Classification of FP according to their type of synthesis and their skills as drug delivery systems.

Systems for drug delivery may be grouped according to Figure 2. An emulsion system can be used for the preparation of various FP systems such as hydrogels, films, spheres and particles. In addition, emulsion polymerization is one of the most common used methods to obtain FP materials. Liposomes and micelles give rise to emulsions. Hydrogels and films are specific structures that are obtained by different polymerization processes and solvent removal, whereas particles are identified as solid forms of FP systems. In the case of dendrimers, they are structures with great advantages, since biomolecules could be incorporated into the branches or in the periphery. In each group there are macro-, micro- and nanosystems, where these last are currently studied as carriers in drug delivery. Some other details of the cited systems are described below.

### 2.1. FP Emulsion Systems as an Enhancement Alternative of Sensitive Molecules

Systems from emulsion formulations of FP have been used as a functional method for the entrapment or the load of sensitive molecules. They are used to encapsulate, to protect molecules and to deliver them to very specific sites within GIT. These systems can also be used as a base to obtain FP structures by solvent separation.

Micelles and liposomes are assembled into emulsions. Micelles are assemblies with a shell and an inner core; they are made up of synthetic block- or graft-copolymers. Due to the hydrophobic core, these systems could act as a reservoir for the encapsulation of hydrophobic drugs and could cross the intestinal barrier after oral administration [21,22]. Their small size (diameter 10–100 nm) allows a prolonged drug circulation time; they also have low toxicity and have high structural stability. Specifically, nanomicelles are now studied for effective drug delivery to carcinogenic cells. Polymer liposomes or vesicles are molecular assemblies of amphiphilic block copolymers or complementary random copolymers. Because of both their hydrophobic and hydrophilic drug moieties, these systems can encapsulate different sensitive molecules.

Chitosan, cellulose derivatives, ethylene glycol and PEG are the most used polymers in emulsions. These systems have demonstrated ability to carry both polar and non-polar molecules, and a better control over their release. Particularly, FP micelles nanomaterials are seen today as one promise in the pharmaceutical area [23]. Mainly, insulin is the most studied biomolecule in FP emulsions on diabetic rats [3]. However, some disadvantages on FP emulsions are their low drug loading efficiency, their poor stability after administration, and their difficulty to move through paracellular membranes; thus, several challenges need to be overcome such as their reduced loading capacity and low physicochemical stability during long term storage [3].

### 2.2. FP Hydrogel and Film Systems as an Enhancement Alternative of Sensitive Molecules

FP hydrogels and films have also been considered as a possibly efficient and convenient way to administer biomolecules. Characteristics of hydrogels and films make these systems models and carriers for oral delivery of sensitive molecules.

**Figure 3:**
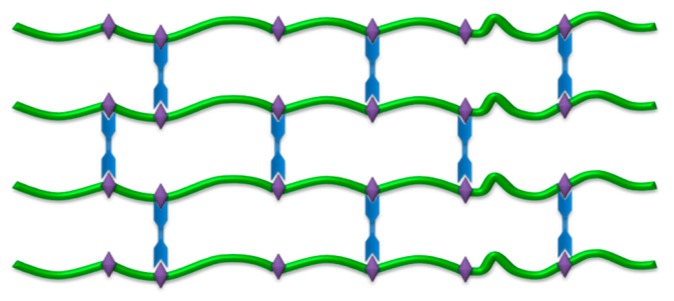
3D hydrogel network showing its macroporous structure. Main polymer chain (green); covalent bonds formed during crosslinking and polymerization process (blue); crosslinking points in the polymer structure (violet).

Hydrogels are permanent or chemical gels stabilized by covalently cross-linked networks that have the capacity to hold water within their porous structure. Figure 3 shows the microporous structure of a hydrogel, which leads to the absorption and desorption of a drug.

Due to hydrogel characteristics, these systems are considered ideal to protect the entrapped active molecules in gastric fluid since they can protect them from degradation by digestive enzymes in the stomach and can also inhibit the activity of Ca^2+^ dependent proteolytic enzymes.

Absorbent and mucoadhesive properties of hydrogels are also exploited for their use as enhancers of sensitive molecules and delivery systems. These also enable prolonged residence time at the absorption site. Covalent attachment of drugs offers greater control over long-term *in vivo* delivery. While appropriate temporal and spatial release profiles can be designed through environmentally responsive drug linkers.

Some FP hydrogels tested for drug delivery, are those based on chitosan and collagen, poly(ethylene glycol) (PEG), poly(*N*-isopropylacrylamide) (PNIPAM) and hyaluronic acid blended with methylcellulose (HAMC), PEG, PNIPAM and HAMC. One disadvantage of these FP systems is their biocompatibility and adhesion of biomolecules, which can alter the polymer structure.

Concerning oral films, these are carrier systems that have mainly been studied for transbuccal drug delivery; however, films are also considered as systems that pass through the GIT without losing activity. In this case, part of the films can be absorbed in the oral cavity, whereas most of the drug is absorbed after swallowing and transition to the GIT. This is possible because films may be carrier of particles that subsequently may achieve the intestinal tract and release the drug in this place [24].

Due to their form and consistency, films may also offer the capacity to permeate trough the intestinal epithelium, allowing the disruption of epithelial barriers, interfering with tight junctions closing mechanism, increasing the fluidity of membranes or by decreasing mucus viscosity.

Cellulose derivatives and PVA are commonly used in films preparation. Sodium alginate, gelatin and pectin, as well as, hypromellose with cationic copolymer based on dimethylaminoethyl methacrylate (DMAEMA), butyl methacrylate (BMA) and methyl methacrylate (MMA) and synthetic copolymers of macrogel-PVA, are also studied to deliver drugs in film forms.

Today, there are a few studies of these FP systems; however, these products can enhance compliance and acceptance. Nevertheless, the small size and form of the oral films have limited their incorporating capacities as active molecules; thus, they must be potent enough at low doses [17].

Finally, both cases, hydrogels and films, can also be designed in nanoscale, getting better the contact area and molecule delivery. Presently these nanosystems, specifically nanofilms could offer great possibilities of application in oral bioavailability of sensitive molecules.

### 2.3. FP Solid Particles Systems as Enhancement Alternative of Sensitive Molecules

Currently, FP solid particles systems are based in microparticles (MPs) and nanoparticles (NPs). However, FP-NPs are the most studied systems in molecules delivery, because they increment their cellular uptake through receptor-mediated endocytosis [25]. NPs are also recognized as delivery enhancers systems that provide an added level of protection from degradation of sensitive molecules and assist co-localized release, improving their therapeutic performance. Several studies of FP solids carriers have demonstrated that is also possible to enhance the molecule stability and performance. This status is achieved by increasing drug solubility, particle wettability and particle porosity to augment drug release. In addition, FP solid particles provide a high specific surface area for adsorption of drug and formation of covalent attachment via amide bond formation. The FP exhibits also good long-term dispersion stability [26].

Biodegradable polymers such as, PLGA, PEG-methyl and ether-block-polylactide (PEG*-b-*PLA) are employed as base material to produce FP-NPs systems. Specifically, hydrophobic polystyrene and chitosan are used as protective material and bioadhesive polymers.

In addition, other studies have proved that FP containing carboxylic acid groups have the ability to protect peptides from the protease enzymes such as trypsin and chemotrypsin. These polymers were proposed to react by the binding of divalent cations (calcium and zinc) to exhibit their enzyme inhibitory effects [27].

Suspension, dispersion, precipitation, multistage, membrane/microchannel emulsification and microfluidic polymerizations are the main techniques to obtain solid particles. Recent advances show also development of FP-NPs systems from layer by layer (LBL). LBL coating of NPs are prepared as strategy to provide a better molecule protection against gastric enzymes, as well as a delayed release, but for prolonged period of time [28]. LBL may be composed of chitosan, alginate, poly(allylamine) (PAA) or PAAc. Moreover, NPs may be obtained by templating mesoporous silica (MS) or with a metallic core to obtain magnetic properties of NPs.

Disadvantages of using nanoparticles as FP systems are related with the low incorporation efficiency of hydrophilic biomolecules, lack of precise control for drug release, tendency of particle aggregation, and the possible accumulation of non-degradable particles in tissues [3]. However, due to their multiples advantages as enhancement systems of sensitive molecules, these FP structures are the most studied in drug delivery.

Currently, the NPs systems are considered as an important study area in physical modification of sensitive molecules to cover of the requirements of cancer diseases.

### 2.4. FP Dendrimers Systems as Enhancement Alternative of Sensitive Molecules

Studies on sensitive molecules may also be associated with FP dendrimer to enhance their oral bioavailability, because they have similar structures to globular proteins or sensitive molecules and thus, are also biocompatibles.

**Figure 4:**
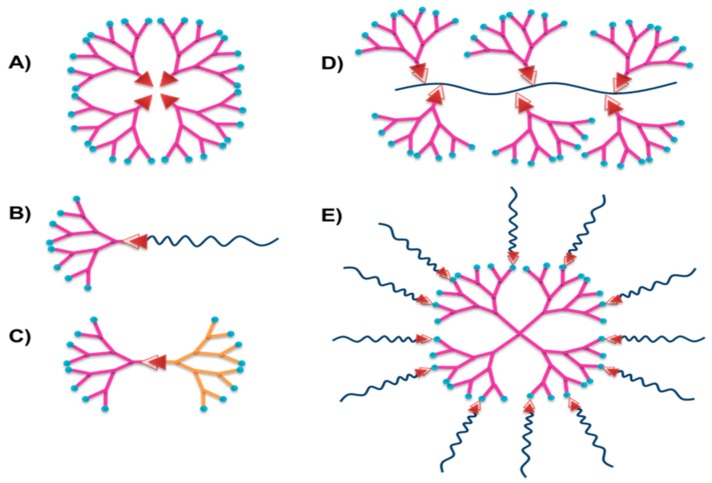
Different architectures of dendritic polymers: (**A**) dendrimer-type; (**B**) linear-dendritic block copolymer; (**C**) Janus dendritic polymer; (**D**) dendronized polymer; and (**E**) dendritic multiarm copolymer. Focal point of dendrimer (Red); branches of dendrimer (pink); terminal groups of dendrimer (blue); main polymeric chain (black); another type of dendron with different functional groups (orange).

Dendrimers are core–shell nanostructures with precise architecture and low polydispersity. Polyvalence of dendrimer provides versatile functionalization with biological receptor sites and functionalization of periphery can give origin at other FP systems with new properties as viscosity and stability [29]. In Figure 4, the chief dendritic polymers used as FP systems are shown, including dendrimer-type, linear-dendritic block copolymer, Janus dendritic polymer, dendronized polymer, and dendritic multiarm copolymer.

The versatility of dendrimers is that, through hydrophobic interactions or through covalent bonding, biomolecules are incorporated in the interior branches or in any place of the dendritic structure [30].

FP dendrimer systems have been studied with the scope to analyze the solubilization of hydrophobic small biomolecules involved in anti-cancer, -depressant, -inflammatory and -microbial applications [31]. This method has also been widely studied for nucleic acid-based therapeutics and other negatively charged therapeutics.

Biomolecules have been associated with FP dendrimers such as poly(amidoamine) (PAMAM), poly(propylene imine) (PPI or DAB), and polyether hydroxylamine dendrimers (PELHAM).

FP dendrimer are considered ideal systems in sensitive molecules delivery, due to capacity for encapsulate and compatibility with gastric fluids, however they should be designed with a better ability to cross the intestinal barrier. Their high cost of preparation is also a disadvantage in drug delivery area.

Further opportunities of these systems as physical enhancement modification of biomolecules is waited, because they offer different properties as viscosity, stability and various advantages of attachment of multifunctional groups to improve the FP system.

An overview on FP mentioned systems, enhancement properties of sensitive molecules and status of application is summarized in Table 1, Table 2 and Table 3. Emulsions, hydrogels, dendrimers, films and solid FP systems make up this report, which is comprised since 2000 to present. FP materials and their properties as permeability, protective enzymatic action and delivery drug control of sensitive molecules, were considered as the highlight data to perform this summary.

Mostly, these characteristics are considered as an indicator for establishing the status of application of molecule and safety and efficacy of a multisource finished pharmaceutical product (FPP).

As can be seen, at present, there are many FP materials integrated in different systems to cover different demands for the bioavailability of sensitive molecules. Principally, they are built to perform physical modifications of these molecules and then to enhance their properties of permeability, protective action of molecule and their release control. FP materials include biopolymers like protein and polysaccharide and synthetic or semi-synthetic polymers; mainly composites FP have been used to prepare various formulations based in chitosan to improve molecule permeability. Thus far, FP-emulsion and FP hydrogels have been the most promising systems to enhance this property; nevertheless, NPs have also been the most studied systems to give response to this stimulus, with some other advantages like protective systems, increase of the half-life of the molecule, high biocompatibility, minimum immunogenicity, site targeting and overcoming the membrane barriers.

The most studied sensitive molecules are insulin, calcitonin and mannitol, and their results as modified molecules by FP systems correspond primarily to *in vitro* studies. However, the possibility of modifying these molecules by FP systems and their response against permeability, protective action and optimal carrier for sensitive molecules, are useful to continue with research on this topic.

## 3. Chemical Modification on the Nature of Sensitive Molecules by FP

Oral bioavailability of drugs can be enhanced by modifying their physicochemical nature by chemical strategies, with the scope to increase membrane permeability, penetration of product in the GIT, protection against degradability and proteolytic stability in this place to be absorbed transcellularly.

Chemical modifications may be performed into specific sites of the molecule or into structure where significant advances have been found under this approach in FP applications.

Table 4 shows actual relevant structural modifications on biomolecules to enhance their physicochemical properties.

The sequence modification of aminoacid terminal is a basic chemical strategy of FP to reduce the enzymatic degradation of active molecules and low bioavailability after oral administration, because of the poor absorption or susceptibility to first pass metabolism. The modification requires identifying the vulnerable sites to perform the substitution with resistant amino acids or specific FP, because any structural alteration may lead to reduction in biological activity.

Techniques of cyclization of sensitive molecules by FP oligopeptides as carriers of sensitive molecules are also different strategies for their chemical modification. In this case, FP, as oligopeptides, are attached to a drug through their amino groups, hence offering the C-terminal carboxyl group a nucleophile to promote intramolecular activation. Conversely, if the drug is attached to the peptide carbonyl, the *N*-terminal amino group will become available to eventually engage in a cyclization-elimination for prodrug activation [93,94].

Adding protein (proteinylation) and molecules as sugars (glycation) and polyols have also been employed for chemical modification of drugs, mainly to develop prodrugs [18]. These modifications affect the structure and maintenance of drug, prevent the loss of the bioactivity and increment the permeability and stability in plasma and the selectivity towards their target site. More details on this chemical modification of drugs can be seen in [8,17].

PEGylation is also another important chemical process in drugs modification. PEGylation consists in the conjugation of one or more PEG molecules to proteins, peptides, non-peptide molecules or particle surface. Reactive amino acids from sensitive molecules include lysine, cysteine, histidine, arginine, aspartic acid, glutamic acid, serine, threonine, tyrosine, N-terminal amino group and the C-terminal carboxylic acid. The most recent advances in biopharmaceutical polymer conjugates show that covalent attachment of molecule with hydrophilic FP can increase the hydrodynamic radius of molecule and thus increase its solubility [95].

In addition, PEGylation improves properties such as stability, sustained absorption, reducing of amount of drug required for therapeutic efficacy, reduced immunogenicity and reduced proteolysis. In general, this modification offers the possibility to tailor the requirements of different sensitive molecules.

Currently, several modified pharmaceuticals by this via, have been approved for their application in the treatment of different diseases and many others are in clinical evaluation; among them are found some pharmaceutical drugs for diabetes [96]. However, these drugs have been modified for administration by other routes, such as parental and intravaginal [97].

Chemical modification of biomolecules has also been associated with FP dendrimers systems via chemical bonding (“prodrug approach”). Targeted delivery is possible via targeting ligands conjugated to the dendrimer surface or via the enhanced permeability and retention effect (EPR) [98].

Scaffolds dendrimers provide extra functional groups for drug conjugation; thus, these FP systems are also used in chemical modifications of biomolecules. In this case, hydrophilic dendrimer conjugates may enhance the solubility of drugs or drug-loaded devices by its inclusion into the pit of the cyclic oligosaccharides [107]. Therapeutic molecules can also directly forms complexes with dendrimers containing counter-charged groups [108]. This method has also been widely studied for nucleic acid-based therapeutics and other negatively charged therapeutic.

Globally, all the reports on chemical modifications of drugs show that FP systems enhance the ability to reduce the frequency of dosing owing to the longer circulating half-life. However, in some cases, it has been found that dosing volumes needed for a longer duration of action can become limiting. In addition, reactions of the immune system to modified molecules can alter it in the long term. Consequently, many of the research based in FP are now found in development, mainly on potential immunogenicity of the polymer.

## 4. Discussion

From the reviewed results about approach enhancement to oral administration of sensitive molecules by different FP systems, significant advances in this topic and relevant data have been identified. Since last decade, several methods have been designed and tested to find an adequate system to deliver active molecules. Protection of sensitive molecules from degradation, prolonged or modified release and increase of their potential activity are the main goals for the development of these methods.

Currently, the contribution of FP systems in physicochemical modification of biomolecules is evident. Assumptions from trials conducted on different FP systems show promising and positive results for their formulation into effective oral sensitive molecules delivery systems.

Nowadays, some sensitive molecules are found in different research status. Some of them have progressed to clinical tests, and thus are likely to become commercially available in the next few years. Some are in a preclinical or discovery stage; meanwhile, others are under development.

This information is available in “Oral Proteins and Peptides Market 2015–2025” [109]. Some relevant data of this report reveal that 76 molecules are currently in different phases of development. Four molecules are in the late clinical test stage (phase III); from these, Oral Octreolin, Ostora and Plecanatide are likely to be made commercially available in 2016. Diabetes stands out among the indicated groups for which different molecules are under study. Nearly 37% of the total number of molecules under research is being developed for diabetic disorders. Other prominent areas include gastric disorders and bone diseases.

Despite these successful results, is necessary to increase the contribution of FP systems on oral bioavailability of sensitive molecules, since at the moment, their contribution has been poor. More studies in vitro and in vivo are necessary to demonstrate the enhancement in the biomolecules and to achieve the clinical phase. In addition, no data on biowaiver of biomolecules were found that show relative bioavailability and/or bioequivalence improvements through the FP systems or a vitro assay on these systems that can replace in vivo studies, to ensure therapeutic equivalence and thus their bioavailability, according BCS.

The study on FP systems as enhancement techniques for biomolecules administration has not been easy, because each molecule requires its own specific condition for stability, solubilization and controlled release immune elimination. Some of the most exposed problems in several reviews are related to permeation [5,13,20,110]. The physicochemical properties of sensitive molecules and conditions of absorption of FP systems have also limited their function [111,112]. *In vitro* results on the properties of biomolecules provide relevant data to predict the *in vivo* performance.

Denaturation at the water/solvent interfaces is another of the major issues that lead to a decrease in the molecule bioactivity occurring during the encapsulation process. In addition, other results have showed that FP nano carriers can reduce the transepithelial resistance [60]; therefore, their potential use for clinical applications is still uncertain [20]. Consequently, some FP systems still cannot be used in oral formats due to their low efficacy. In addition, some of them are applied and commercialized today, and reports on clinical studies are scarce [96]. Accordingly, more studies are necessary on the aggregation form of the molecule, as well as clinical tests. Furthermore, the biodegradability of FP must be considered for the design of the delivery systems, due to problems associated with long-term biocompatibility.

A restrictive assessment on toxicity of FP systems is also necessary, as well as their effects to the intestinal epithelial cells [113]. FP may induce cell damage, mainly in buccal and intestinal cells which may result in acute and, possibly, chronic toxic effects [114]. Impact of their toxicity can be seen in ulcerations of the intestinal epithelium and erosions due to the ingestion of pharmaceuticals [115].

## 5. Conclusions

FP systems have an important contribution in the strategies for oral administration of sensitive molecules. These systems are used in physical and chemical modification of drugs to increase their oral bioavailability and to remain in an intact form through GIT for their release.

Nowadays, several reports have demonstrated that the efficacy of these molecules could be improved by inclusion of FP systems and some of them are now available for consumption. However, due to physicochemical characteristics of sensitive molecules, many challenges still exist to enable their optimal oral supply. In addition, the higher introduction of these molecules as biopharmaceuticals also demands a high amount of studies in this area, and requires fast results of candidates entering clinical studies in a wide variety of therapeutic categories. Furthermore, new oral formulations based on FP systems are necessary to enhance the bioavailability of sensitive molecules.

## Figures and Tables

**Table 1:** FP systems with permeability enhancement on sensitive molecules.

System	FP	Sensitive molecule	References	Application trials
**Micelles**	*N*-trimethyl chitosan (TMC 60, TMC 40 substitution grade)	Hydrophilic [^14^C]-mannitol	[32]	*In vitro*, Permeability of drug by Transepithelial electrical resistance (TEER) on Caco-2-cell
**Micelles**	Labrasol-d-a-tocopheryl-PEG 1000 succinate (TPGS)	Vancomycin hydrochloride (VCM)	[33]	*In vitro*, Permeability by plasma drug concentrations in rat ileum
**Microtablets**	Chitosan-4-thiobutylamidine (TBA)-BBI-chitosan elastatinal-glutathione	Salmon calcitonin (cST)	[34]	*In vitro*, Permeability by the release profile of drug. *In vivo*, Plasma calcium level in rats
**Microemulsion**	SMEDDS-Labrasol	Mannitol	[35]	*In vitro*, TEER measurement on Caco-2-cell.
**Film**	PVA-elastoid^®^ E35H-PPR.PVP	Lidocaine Hydrochloride	[36]	*In vitro*, Transdermal film in vertical Franz-type diffusion cells.
**Film**	Pectin-chitosan	Paracetamol	[37]	*In vitro*, Permeability by release of drug on fresh porcine small intestine
**Emulsion**	Chitosan-glyceryl monostearate (GM)	Enoxaparin	[38]	*In vitro*, Permeability by absorption of drug on intestinal mice
**Nanoparticles**	Chitosan-PLA*-co-*glycolide)-silicon onto of hydroxyl-propylmethyl-cellulose- acetyl-succinate	Peptide Glucagon-like peptide-1	[39]	*In vitro*, Permeability by release of drug in simulated fluids, SGF and SIF
**Emulsion**	Poly(*N*-vinylacetamide*-co-*acrylic acid)-d-octaarginine,	Protein (pGFP-C1), β-galactosidase and bovine serum albumin (BSA)	[40]	*In vitro*, Permeation drugs in HeLa-cells
**Nanoparticles**	*N*-trimethyl-TMC-(PLGA)	Insulin	[41]	*In vitro*, Permeability by release of drug in simulated gastric fluid (SGF) and simulated intestinal fluid (SIF). *In vivo*, Intestinal mucoadhesion in male Kumming mice.

**Table 2:** FP systems with protective enzymatic degradation as enhancement of sensitive molecules.

System	FP	Sensitive molecule	References	Application trials
**Capsules/tablets**	Sodium carboxy-methyl cellulose (Na-CMC)- Bowman–Birk inhibitor conjugate	Insulin	[42]	*In vitro*, Protective effect of degradation against intestinal proteases
**Microcapsules/Tablets**	Sodium carboxymethylcellulose (Na-CMC)-Bowman-Birk inhibitor coated with a polymethacrylate	Insulin and mannitol	[43]	*In vitro*, Protective effect against intestinal enzymes and release profile. *In vivo*, Glucose levels of diabetic in mice.
**Microspheres**	Poly(vinyl alcohol)*-graft-*poly(lactic*-co-*glycolic acid), PVA*-g-*PLGA	Bovine serum albumin, ovalbumin, cytochrome c and FITC-dextran	[44]	*In vitro*, Protective effect against intestinal enzymes and release profile.
**Nanoparticles**	PNC of diblock PEG-PLGA	Catalase enzyme	[45]	*In vitro*, Protective effect against proteolysis.
**Nanoparticles**	Triglyceride nanostructures-chitosan-PEG	Salmon calcitonin (cST)	[46]	*In vitro*, Protective effect by TEER measurement on cell monolayer Caco-2. *In vivo*, Serum calcium levels in rats
**Nanoparticles**	Vitamin B12-dextran	Insulin	[47]	*In vitro*, Protective effect against intestinal enzymes. *In vivo*, Glucose measure in diabetic rats
**Block copolymer micelles**	Poly(ethylene oxide)–poly(propylene oxide)	Efavirenz (EFV)	[48]	*In vitro*, Protective effect against intestine-mimicking. *In vivo*, Plasma concentration in different male Wistar rats.
**Nanocomplexes**	PAA-cetyl or cholesteryl choloroformate pendant groups	Bovine Insulin	[49]	*In vitro*, Degradation of drug by intestinal enzymes on Caco-2 cells
**Nanospheres**	Cationic-β cyclodextrin polymers (CPβCDs)	Insulin	[50]	*In vitro*, Degradation of drug against intestinal enzymes into SGF and SIF.
**Nanocapsules**	Sodium caseinate and starch	Fish oil powders protein	[51]	*In vitro*, Degradation of drug by intestinal enzymes.
**Nanoemulsions**	Bovine Lactoferrin stabilized with poloxamers (PEO-PPO-PEO)	Lactoferrin	[52]	*In vitro*, Protective degradation of drug and antimicrobial trials.
**Microcapsules**	PLGA	Ovalbumin and tetanus toxoid (TT),	[53]	*In vitro*, Degradation of drug by intestinal enzymes.
**Emulsion**	Chitosan-thioglycolic acid (CS-TGA)	Salmon calcitonin (cST)	[54]	*In vitro*, Protective degradation of drug into porcine intestine mucus. *In vivo*, Release of drug in male Sprague-Dawley (SD) rats
**Microspheres**	PLGA	Immuno-globulin G	[55]	*In vitro*, Preserving the integrity of the encapsulated antibody
**Nano-particles Ritonavir (RTV)**	PEG- PVA- Pluronic^®^ F68, ^®^ F127- PVP K30- HPC and/or HPMC	Cytochrome P4503A4 (CYP3A4) and P-glycoprotein (P-gp)	[56]	*In vitro*, Protective degradation of drugs into cells, HepG2, Caco-2, THP-1, A-THP-1, and CEM

**Table 3:** FP systems with absorption and delivery enhancement of sensitive molecules.

System	FP	Sensible Molecule	References	Application trials and results
**Nanoparticles**	PVAL*-graft-*PLGA	Tetanus Toxoid (TT)	[57]	*In vitro*, Release of drug into intraperitoneal mice.
**Nanoparticles**	PLGA-mPEG	Cisplatin	[58]	*In vitro*, Release of drug in SGF. *In vivo*, Effect of drug in female BALB/c mice.
**Film**	Ethyl cellulose (EC)-polyvinyl pyrrolidone (PVP)-dibutyl phthalate (DBP)	Propanolol Hydrochloride (PPL)	[59]	*In vitro*, Release of drug in SGF.
**Nanoparticles nanocapsules**	Chitosan-oil nanodroplets	Salmon calcitonin (cST)	[60]	*In vitro*, Release of drug in SGF. *In vivo*, Calcemic levels observed in rats
**Nanoparticles**	PLA-poly(d,l-lactide*-co-*glycolide acid) (PLGA)	Porcine Insulin	[61]	*In vitro*, Release of drug in SGF. *In vivo*, Glucose evaluation in diabetic rats.
**Nanoparticles**	Polyester-polycationic polymethacrylate	Tinzaparin	[62]	*In vitro*, Release of drug into intestinal rabbit. *In vivo*, Anticoagulant effect in rabbit
**Dispersed nanoparticles in a film**	DOCA (HD) dispersed in polyurethane	Heparin	[63]	*In vitro*, Release of drugs into endothelial cell (HUVEC) *In vivo*, Anti-proliferative effects on old male C3H/HeN mice
**Nanoparticles**	PLGA-NP	Cyclosporine	[64]	*In vitro*, Release of drug into heparinized blood from SD rats. *In vivo*, Effect of drug in male SD rats.
**Vesicles**	PLA*-b-*Pluronic*-b-*PLA-F127-PLA	Bovine insulin	[65]	*In vitro*, Release of drug in SGF. *In vivo*, Hypo-glycemic effect in Kumming diabetic mice.
**Nanoparticles**	Chitosan-triethylchitosan (TEC)-dimethyl-ethylchitosan (DMEC)	Insulin	[66]	*In vitro*, Release of drug for 5 h in SGF.
**Microspheres**	PLGA RG 503H	Neurotrophic factor (GDNF) glycosylated	[67]	*In vitro*, Release of drug into cell neurite.
**Tablets and pellets**	HPMC-CMC-EC	Metoclopramide hydrochloride (MCP)	[68]	*In vitro*, Release of drug in SGF.
**Microparticle**	Chitosan-alginate-pectin	Bovine serum albumin (BSA)	[69]	*In vitro*, Release of drug in SGF.
**Microspheres**	(DE) pectin-calcium-chitosan PCaC	Mangiferin	[70]	*In vitro*, Release of drug in both SGF and SIF.
**Microspheres**	Chitosan-glutaraldehyde	Insulin	[71]	*In vitro*, Release of drug in SGF. *In vivo*, Glucose level and powerful therapeutic effects in SD rats
**Dendrimers**	Polyamidoamine (PAMAM)	5(6)-(CF), fluorescein isothiocyanate-dextrans (FDs), calcitonin and insulin	[72]	*In vitro*, Release of drugs into rat small intestine.
**Hydrogel**	Poly(*N*-isopropylacrylamide*-co-*propylacrylicacid*-co-*butylacrylate)	Fibroblast growth factor (bFGF)	[73]	*In vitro*, Delivery of drug in SGF. *In vivo*, Cardio-vascular function after 28 days in old Fischer rats.
**Nanoparticles**	Chitosan-hydroxypropyl methylcellulose phthalate (HPMCP)	Insulin	[74]	*In vitro*, Release profiles in SGF without enzymes. *In vivo*, Mucoadhesion studies in male Wistar rats.
**Nanoparticles**	Poly(lactic*-co-*glycolic acid)	Salmon calcitonin (sCT)	[75]	*In vitro*, Release of drug into SGF and SIF *In vitro*, Plasma calcium level in female SD rats.
**Hydrogel**	Chitosan-β–glycerophosphate (β–GP)	Desferroxamine (DFO)	[76]	*In vitro*, Release of drug into endothelial cell (HUVEC).
**Nanoparticles**	Chitosan	Anticancer Gemcitabine (GC, 2′,2′difluorodeoxycytidine)	[77]	*In vitro*, Release of drug into HT-29 cell.
**Hydrogels**	Poly(acrylamide)*-graft-κ-*carrageenan (PAAm*-g-*CG) and sodium alginate (SA)	Ketoprofen	[78]	*In vitro*, Release of drug into rat stomach.
**Spherical magnetic**	Pectin-chitosan	Diclofenac sodium (DS)	[79]	*In vitro*, Magnetically guided targeted drug delivery SGF.
**Nanoparticles**	Polybenzofulvene derivative (poly-6-MOEG-9-BF3k)	Leuprolide	[80]	*In vitro*, Drug delivery on epithelial cell; 16 HBE). *In vivo*, Effect of drug on male Winstar rats.
**Tablets**	Acetylated moth bean starch (AMBS)	Lamivudine	[81]	*In vitro*, Evaluation of controlled release of drug in stomach of white albino rabbits.
**Nanoporous peptide particles**	Polypeptide poly(l-glutamic acid) (PGA)	Brain-derived neurotrophic factor (BDNF)	[82]	*In vitro*, Release of drug in SGF.
**Hydrogel**	Conjugated linoleic acid coupled with pluronic F-127 (Plu-CLA)	Docetaxel	[83]	*In vitro*, Release of drug into human gastric cancer cells. *In vivo*, Anti-tumor effect on peritoneal gastric cancer metastasis in male BALB/c nude mice.
**Nanoparticles**	CpG-loaded onto poly(l-glutamic acid) (PGA)	Oligonucleotid CpG	[84]	*In vitro*, Release of drug into peripheral blood mononuclear cells (PBMC’s).
**Microparticles**	Trimethyl-chitosan (TMC)-PEG-PEGDMA-MAA	Interferon-β(INF-β)	[85]	*In vitro*, Drug release in SGF. *In vivo*, Effect of drug in rabbits.
**Nanoparticle (SLN)**	Glyceryl monostereate-Tween 80	Efavirenz (EFV)	[86]	*In vitro*, Release of EFV in SGF.
**Nanospheres**	Alginate-silica	Indomethacin (IND)	[87]	*In vitro*, Drug delivery in SGF.
**Particles**	PLGA-PEG	Vascular endothelial growth factor (VEGF)	[88]	*In vitro*, Release of drug in SGF.
**Hydrogel (IPN)**	Poly(aspartic acid); KPAsp Carboxymethyl chitosan	Salicylic acid	[89]	*In vitro*, Release of drug in SGF.
**Film**	Tamarind Seed Polysaccharide (TSP)-β (1→4)-d-glucan mostly	Nystatin	[90]	*In vitro*, Release of drug in SGF.
**Nanoparticles**	Poly(vinylpyrrolidone) (PVP)	Ketoprofen	[91]	*In vitro*, Drug delivery into a PVP matrix of spatially confined microcontainers.
**Nanoparticles**	Acetylated corn starch	Ciprofloxacin (CFx)	[92]	*In vitro*, Release of drug in SGF.

**Table 4:** Modification of the chemical nature of biomolecules with FP systems.

Enhance modification by FP	Biomolecule	Enhancement action	References	Application trials
Monomeric and dendrimeric tetrabranched form by residue substitution	Synthetic antibacterial peptide	Stability to blood proteases	[99]	*In vitro*, Antimicrobial activity against a panel of gram-negative bacteria
Substitution of two polycationic lipophilic-core carbohydrate-based dendrons 2a-b and five polycationic lipophilic-core peptide dendrons 3–6, containing aminoacid terminal residues	Heparin (LMWH)	Absorption molecule in intestinal trials	[100]	*In vitro*, Absorption in simulated intestine. *In vivo*, Effect of drug in LMHW rats.
PAMAM and PPI dendrimers by introducing functional groups	Methotre-xate sodium	Stability of molecule	[101]	*In vitro*, Release of molecule in simulated gastric fluids.
The 3483 Da peptide glucagon PEGylated to amino acid residue Lys12 (gluc-PEG-L) with branched PEG chain of 2200 Da (gluc-PEG-B)	Glucagon	Increase in adsorbing per unit surface area rate	[102]	*In vitro*, Release of molecule in simulated gastric fluids.
Functionalized nanoliposomes with synthetic coupling of the peptide (Lys-Val-Leu-Phe-Leu-Ser)	Ligand-functionalized nanoliposomes	Absorption of molecule	[103]	*In vitro*, Release in SGF. *In vivo*, Effect on neuronal cell in rats.
PEGylated derivatives of Bac7 (1-35)	Peptide Bac7(1-35)	Protective effect and stabilization	[104]	*In vitro*, Protective action against S*. typhimurium*
Poly(ethylene glycol)-l-asparaginase (PEG–ASNase), Poly(*N,N*-dimethylaminoethyl methacrylate) (PAMA), PEG-*b*-PAMA	l-Asparaginase (l-Asn)	Stabilization of molecule	[105]	*In vitro*, Protective degradation against intestinal enzymes.
12 triterpenic PEGylated amine derivatives	Oleanolic and maslinic acids	Cytotoxicity of molecule	[106]	*In vitro*, Apoptotic effects on cancer-cell lines (B16single bondF10, HT29, and Hep G2)
